# Smenospongine, a Sesquiterpene Aminoquinone from a Marine Sponge, Induces G1 Arrest or Apoptosis in Different Leukemia Cells

**DOI:** 10.3390/md20080023

**Published:** 2008-08-26

**Authors:** Dexin Kong, Shunji Aoki, Yoshihiro Sowa, Toshiyuki Sakai, Motomasa Kobayashi

**Affiliations:** 1 Graduate School of Pharmaceutical Sciences, Osaka University, Yamada-oka 1–6, Suita, Osaka 565–0871, Japan; 2 Department of Preventive Medicine, Kyoto Prefectural University of Medicine, Kawaramachi-Hirokoji, Kamigyo-ku, Kyoto 602–8566, Japan

**Keywords:** Smenospongine, G1 arrest, apoptosis, leukemia cells, p21, Rb

## Abstract

Smenospongine, a sesquiterpene aminoquinone isolated from the marine sponge *Dactylospongia elegans*, was previously reported by us to induce erythroid differentiation and G1 phase arrest of K562 chronic myelogenous leukemia cells. In this study, we investigated the effect of smenospongine on the cell cycles of other leukemia cells, including HL60 human acute promyelocytic leukemia cells and U937 human histiocytic lymphoma cells by flow cytometric analysis. Smenospongine induced apoptosis dose-dependently in HL60 and U937 cells. The smenospongine treatment increased expression of p21 and inhibited phosphorylation of Rb in K562 cells, suggesting the p21-Rb pathway play an important role in G1 arrest in K562 cells. However, the p21 promoter was not activated by the smenospongine treatment based on a luciferase assay using the transfected K562 cells. Smenospongine might induce p21 expression via another mechanism than transactivation of p21 promoter.

## 1. Introduction

Chronic myelogenous leukemia (CML) is a hematopoietic stem cell cancer caused by the Bcr-Abl tyrosine kinase which arised from Philadelphia chromosome (Ph) translocation [1,2]. The only known curative therapy for CML is allogeneic bone marrow transplant (BMT). However, the treatment-related toxicity is severe, with reported mortality at about 30% [3]. A selective inhibitor of Bcr-Abl, imatinib mesylate (Glivec^®^), was developed as the first molecule-targeted anticancer drug [4]. Within the last decade, imatinib has proven to be highly efficacious for treatment of CML. However, with the widespread use of this drug, the resistance in some patients has been well reported, which is usually caused by mutations within Bcr-Abl [3,5,6]. Therefore, development of alternative drugs to imatinib for CML treatment has been expected. In addition, since Bcr-Abl is only present in CML and about 20% of cases with acute lymphoblastic leukemia, imatinib shows its limitations on other leukemia diseases. On the contrary, it was reported that FK228, a histone deacetylase (HDAC) inhibitor, was indicated to be therapeutically effective in treating not only CML, but also AML (acute myelogenous leukemia) and lymphocytic leukemia [7–9]. Consequently, there is great interest in discovering and developing similar antiumor agents showing therapeutic effects on all leukemias.

As a part of our discovery of new anticancer drug candidate from natural resources, we have been searching for new differentiation-inducing substances or cell cycle inhibitors for CML cells [10,11]. With the guidance of a bioassay method for erythroid differentiation of K562 CML cells, we isolated smenospongine, a sesquiterpene aminoquinone, from the Indonesian marine sponge *Dactylospongia elegans*. Smenospongine induced cell cycle arrest at the G1 phase and increased p21 expression [12,13]. One question, whether smenospongine shows the same effect on other leukemia cells or not, remained unclear. We recently investigated the effect of smenospongine on other leukemia cells such as HL60 and U937 by cell cycle analysis and further investigated the mechanism for the G1 arrest in K562 cells.

## 2. Results and Discussion

### 2.1. Effect of smenospongine on cell cycle distribution in K562, HL60, and U937 cells

**Figure 1:**
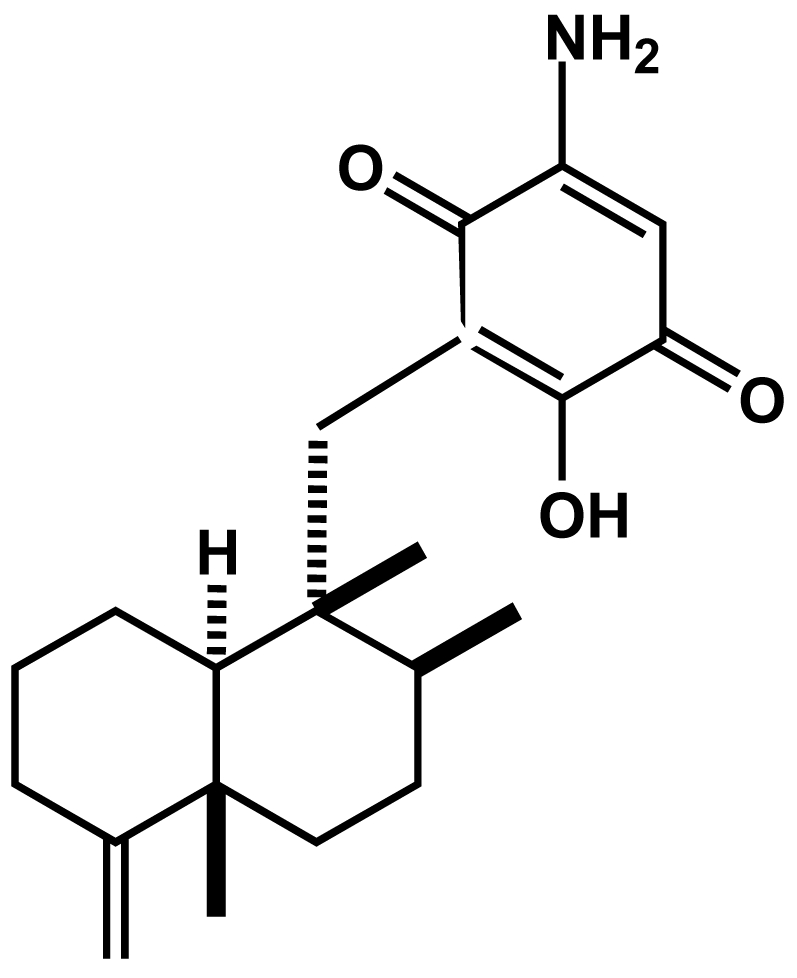
Chemical structure of smenospongine

**Figure 2:**
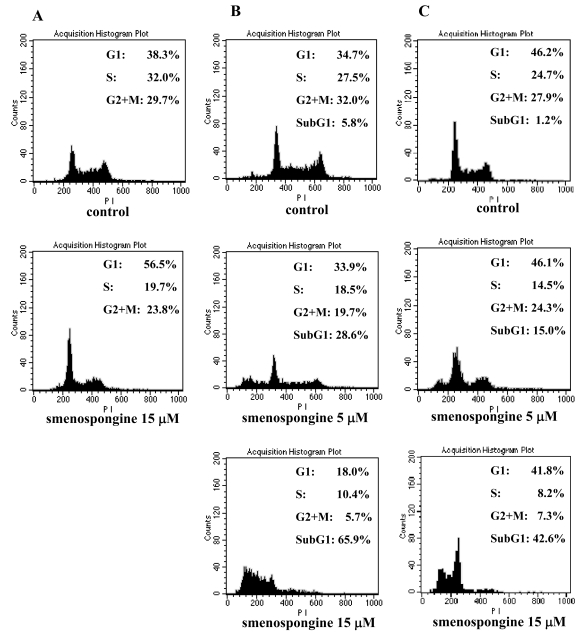
Effect of smenospongine on cell cycle distribution of K562, HL60, and U937 cells. A suspension of K562 (**A**), HL60 (**B**), and U937 (**C**) cells (2 × 10^5^ cells/2ml/well) was incubated in the presence or absence of various concentrations of smenospongine in an 8-well plate under a 5% CO_2_ atmosphere at 37°C, respectively. After 24 h, the cells were collected and dyed with DNA-Prep Reagents Kit for cell cycle analysis. The analysis was carried out with flow cytometer (λex = 493 nm, λem = 630 nm) and quantified by ModFit Software.

Our previous study showed that smenospongine (Figure 1) induced G1 arrest in K562 cells [12]. To investigate whether this compound has the same effect on other leukemia cells or not, cell cycle analysis for HL60 and U937 cells together with K562 cells was carried out. Various concentrations of smenospongine as DMSO solution were added to the randomly cultured K562, HL60, and U937 cells, respectively. After 24 h, the cells were collected for cell cycle analysis. As a result, smenospongine induced G1 phase arrest in K562 cells at 15 μM concentration (Figure 2A), which is consistent with our previous report [12]. G1 arrest was not found in HL60 and U937 cells, but sub-G1 accumulation, which indicates apoptosis, was observed. And the induction of apoptosis was shown to be dose-dependently (Figure 2B, 2C). In order to check whether this is attributed to the difference of the sensitivity for individual cells or not, we further analyzed with lower doses of smenospongine which did not induce apoptosis in both cells. However, no G1 accumulation was detected yet in both cells (data not shown). This suggests that smenospongine might affect different leukemia cells via the respective mechanism.

### 2.2. Effect of smenospongine on expression of p21 and phosphorylation of Rb

**Figure 3:**
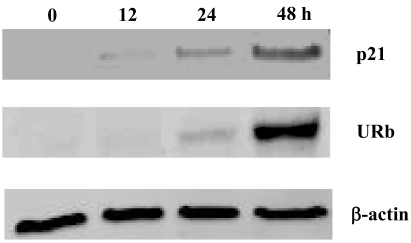
Effect of smenospongine on expression of p21 and phosphorylation of Rb. K562 cells (1 × 10^6^ cells/10ml) were incubated with 15 μM smenospongine for the indicated time periods. The cell lysate was prepared and applied to 4–20% SDS-PAGE. After being transferred to PVDF membrane, the blots were exposed to anti-Cip1/WAF-1/p21, anti-human underphosphorylated Rb (URb) or anti-β-actin antibody and then anti-mouse IgG HRP-conjugated antibody. The bound antibodies were visualized using an Enhanced Chemiluminescence (ECL) system.

To demonstrate that the G1 phase arrest by smenospongine is attributed to p21 induction and inhibition of the phosphorylation of Rb, which located in the downstream of p21 and is known to play a key role in cell cycle progression from G1 to S phase, p21 expression and phosphorylation of Rb in K562 cells were examined. A 15 μM concentration of smenospongine was added to K562 cells. The cells treated for different length of time were collected, and the respective cell lysate was prepared for Western blotting analysis. The expression of p21 was detected after 12 h and increased in a time-dependent manner until 48 h (Figure 3). Accordingly, the underphosphorylated Rb protein (URb) was also detected after 24 h and highly increased until 48 h, indicating that the phosphorylation of Rb protein was inhibited by the smenospongine treatment for 24 to 48 h. This result suggests that the p21-Rb pathway might play an important role in the G1 phase arrest in K562 cells induced by smenospongine.

### 2.3. Effect of smenospongine on activation of p21 promoter

**Figure 4:**
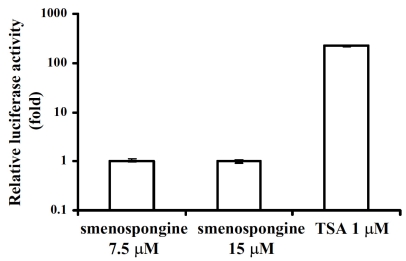
Activation of p21 promoter by smenospongine and TSA in the transfected K562 cells. K562 cells were transfected with pWWP, which is a human wild-type p21 promoter-luciferase fusion plasmid, by DEAE-Dextran Transfection Kit. The cells were incubated at a density of 5 × 10^4^ cells/ml in a 12-well plate for 24 h. 125 ng of pWWP in DEAE-Dextran was added to perform transfection for 15 min. The transfected cells were further incubated for 24 h and then treated by DMSO, smenospongine or TSA for another 24 h. Finally, the cells were collected and the luminescence was measured by using MICRO LUMAT Plus LB96V. The activation of p21 promoter was evaluated by the relative light intensity compared with that of the control (cells treated with DMSO only).

Since K562 cell is known as p53-negative cell [14], the p21 induction can be considered as p53-independent. Furthermore, the induction of p21 mRNA expression by smenospongine was also observed by RT-PCR analysis (data not shown). This suggested the possibility that the p21 expression was induced by trans-activating p21 promoter. In order to investigate whether smenospongine can trans-activate p21 promoter or not, we transfected K562 cells with pWWP, a human wild-type p21 promoter-luciferase fusion plasmid. Luciferase assay was carried out with the transfected cells after DMSO, smenospongine or trichostatin A (TSA) treatment. TSA was used as a positive control, since it was previously reported to activate p21 promoter [15,16]. As a result, the luciferase activity of the smenospongine-treated K562 cells was not significantly increased, while the TSA-treated cells showed about 200 fold enhancement over those DMSO-treated, indicating that smenospongine can not activate p21 promoter (Figure 4). Therefore, we postulate that smenospongine might act by stabilizing p21 mRNA to increase mRNA and protein expression and lead to G1 arrest in K562 cells via p21-Rb pathway. The mechanism of smenospongine to induce apoptosis in HL60 and U937 cells, including effect on p21 expression, is under investigation.

## 3. Conclusions

Smenospongine, a sesquiterpene aminoquinone isolated from the marine sponge of *Dactylospongia elegans*, induced G1 arrest in K562 cells, while induced apoptosis in HL60 and U937 cells. P21-Rb pathway is considered to play an important role in its effect of the G1 arrest in K562 cells.

## 4. Experimental Section

### 4.1. Materials

4–20% gel cassette was purchased from Daiichi Pure Chemicals Co., Ltd. DNA-Prep Reagents Kit was from Coulter Co. RPMI 1640 and DMEM Medium were from Nissui Pharmaceutical Co., Ltd. Polyvinylidene fluoride (PVDF) membrane was from Amersham Pharmacia Biotec. UK Ltd. Other reagents were from Sigma Co., Ltd. or Wako Pure Chemical Industries Co., Ltd unless stated specifically.

### 4.2. Cell culture

K562, HL60, and U937 cells, which were provided by RIKEN Cell Bank, were routinely maintained in the RPMI 1640 (for K562 and U937) or DMEM (for HL60) medium supplemented with 10% fetal bovine serum, kanamycin (100 μg/mL) and glutamine (0.44 mg/mL) at 37°C in a humidified atmosphere containing 5% CO_2_.

### 4.3. Antibodies

Anti-Cip1/WAF-1/p21 antibody was purchased from Upstate Biotechnology Inc.; anti-human underphosphorylated Rb antibody was from BD Biosciences; anti-β-actin antibody was from Sigma Co., Ltd; anti-mouse horseradish peroxidase (HRP) conjugated antibody was from Nacalai Tesque Inc.

### 4.4. Isolation and identification of smenospongine

Smenospongine was isolated from the marine sponge *Dactylospongia elegans* (collected in Indonesia in 2001) as described previously [12]. Briefly, the dried marine sponge was cut into pieces and extracted with methanol. The resulting methanol extract was subjected to solvent partition to give hexane, 90% methanol, *n*-BuOH, and H_2_O soluble portions. The 90% methanol soluble portion was separated by repeated SiO_2_ column, reversed-phase column, and high-performance liquid chromatography (HPLC) to obtain smenospongine. The structure of smenospongine was identified by comparison of the mass and NMR data with those reported [17].

### 4.5. Flow cytometric analysis of cell cycle

The cell suspension (2 × 10^5^ cells/2 ml/well) of K562, HL60 or U937 cells was placed in an 8-well plate and incubated for 24 h at 37°C under a 5% CO_2_ atmosphere. The DMSO solution of the testing sample (10 μl) was added and further incubated for 24 h. The cells were harvested and collected, followed by staining with DNA-Prep Reagents Kit for 20 min. Then, the supernatant was removed by centrifugation, and the cell pellets were re-suspended in 500 μl of D-PBS (−). After being filtered with a 40–μm nylon mesh filter, the cell suspension was available for cell cycle analysis by flow cytometer (λex = 493 nm, λem = 630 nm). The analysis result was quantified by ModFit software (Verity Software, Topsham, ME).

### 4.6. Western blotting analysis

The cell suspension (1 × 10^6^ cells/10 mL) of K562 cells was incubated in a flask together with 15 μM of smenospongine for the indicated length of time under a 5% CO_2_ atmosphere at 37°C. The cells collected by centrifugation (1000 g for 3 min at 4°C) were washed with cold PBS and treated with lysis buffer (50 mM Tris-HCl, pH7.2; 1% NP-40; 0.25% sodium deoxycholate; 150 mM NaCl; 1 mM EDTA; 1 mM PMSF; 1% proteinase inhibitor cocktail) to furnish a cell lysate. Protein assay was carried out using a Bio-Rad protein assay kit. After boiling at 95°C for 5 min in the sample buffer (0.125 M Tris-HCl (pH 6.8), 10% 2-mercaptoethanol, 4% SDS, 10% sucrose, 5% bromophenol blue), the equal amounts of protein were subjected to SDS-Polyacrylamide gel electrophoresis (SDS-PAGE) and then transferred to PVDF membrane. The membrane was blocked with 5% milk TBS (Tween PBS) and exposed to anti-Cip1/WAF-1/p21, anti-human underphosphorylated Rb, or anti-β-actin antibody and then anti-mouse IgG HRP-conjugated antibody. The bound antibodies were visualized using an Enhanced Chemiluminescence (ECL) system.

### 4.7. Transfection of K562 cells and luciferase assay

K562 cells were transfected with a human wild-type p21 promoter-luciferase fusion plasmid, pWWP, by DEAE-Dextran (CellPhect Transfection Kit, Amersham Pharmacia Biotech). The cells were incubated at a density of 5 × 10^4^ cells/mL in a 12-well plate for 24 h. The 125 ng of the reporter plasmid DNA, pWWP, in DEAE-Dextran was added to perform transfection for 15 min. The transfected cells were further incubated for 24 h followed by DMSO, smenospongine or TSA treatment for another 24 h. Finally, the cells were collected for luciferase assay, which was carried out by use of the luciferase assay system (E1501, Promega) as described previously [18]. The luminescence was measured by using MICRO LUMAT Plus LB96V (BERTHOLD) and WING LOW software. The activation of p21 promoter was evaluated by the relative light intensity compared with that of the control (cells treated with DMSO only) [18].
